# Annotations

**Published:** 1893-06-10

**Authors:** 


### ANNOTATIONS.

**A Reminder.** LAST week we published our special "Hospital Sunday Number." We published early in order that there might be ample time for readers to see what hospitals had to say for themselves from various points of view. What hospitals have really to say for themselves is one thing; what we said for them last week is quite another. Although we made out as good and varied a case as our capabilities and resources would permit, we are but too conscious that the hospitals might with complete justification make serious complaint of their advocates. the truth is, that nobody understands, nobody can be made to understand, the nature and extent of the work of the London hospitals, except by going to see it: and if any outsider wishes to see it in some measure as it is, he must be prepared to spend at least a year in going from hospital to hospital. Even then he will only have obtained a brief and cursory glance. There are in London 136 hospitals and dispensaries, all told, and of even the least satisfactory and the least well managed, it may be said with truth that they do good and necessary work, and that they waste no money in the doing of it. Some day, perhaps in fifteen or twenty years from the present time, it will dawn upon the British public that they have rare servants in hospital doctors and nurses, and admirable institutions in the hospitals. When that day comes money will not need to be drawn out of reluctant pockets like an unwilling fox from his earth; it will fly out of itself as if compelled by a magician's wand. What a pity we cannot anticipate that fortunate time and have a foretaste of it next Sunday! Perhaps we shall! We at any rate ive in lively hope.

**"A Common Fault."** IN his valedictory address as President of the Clinical Society, Sir Dyce Duckworth ventured a few words of remonstrance to the members which may very properly be applied to the medical profession generally. "It is a common fault nowadays," said Sir Dyce, "to try to agree with everybody, to have too little courage of one's convictions, and to be afraid to speak out. We need a little more friendly friction. This would be good for us, and I make bold to urge it." It has been said of a good many members of the medical profession that "they dare not call their souls their own"; and to this it has been wittily responded that perhaps the reason of their quite abnormal timidity is that "they have not got any souls." Some medical men have long stood in terrified awe of the Royal College of Physicians, of which Sir Dyce Duckworth is the distinguished treasurer. But these will now, no doubt, cast away their fears. They have long known that the President of that august body, Sir Andrew Clark, is a liberal-minded man; and now that Sir Dyce Duckworth has declared himself on the side, not only of free, but even of bold speech, the timid will pluck up heart and look about in search of their shivering souls. But seriously, we are very glad that a little encouragement has been given in high places to the fearful and the weak. The work of the medical profession is of a very responsible character, and its members are exceedingly liable to be falsely accused by the public. When to this is added the eager talebearing of interested medical competitors, and a too great readiness on the part of medical leaders to assume that "where there is smoke there must be fire," forgetful of the fact that dust or vapour may look like smoke in the distance, the life of the practitioner who is of a more independent mind than some of his fellows becomes decidedly trying. There have been men who would have adorned the ranks of the Royal College of Physicians who have never sought its membership because of the fear they have felt lest its narrow and timid liberties should damp their courage and immure their candour in a prison. It is quite credible that some of the Fellows of the College of Physicians to day would have been greater, worthier, and happier men if they had never known its fellowship. A doctor should not sell his soul, if he happens to have one, either to the College of Physicians or to the medical profession. He must be a man first and a medical practitioner afterwards.

**Ridiculous Fellows of the Royal Society.** Do the Fellows of the Royal Society sometimes devote a little time to the consideration of their own *raison d'être?* It seems hardly likely. The Royal Society, we believe, was established, and is maintained to further the advance of natural knowledge; of science, in short. Many of the fellows, and more particularly those of the professorial element, seem to imagine that it was founded in order to confer scientific decorations upon them. But this very idea of theirs shows how utterly wanting in the true *esprit* of science professed scientists may be. Sir Henry Howarth was proposed this year for election among the fifteen who are annually canonised and beatified by the Pope and the cardinals of science. What were Sir Henry's claims? He has published, and probably at a loss, eight large volumes on scientific subjects; he has contributed no fewer than ninety papers on geology and allied branches of physical science to various scientific journals; and he has been a frequent contributor to the columns of the *Times* on similar subjects. Now, whatever may be the value of his eight volumes, any sensible man knows that his ninety papers published by scientific journals, and his frequent contributions to the *Times*, must have been up to a very reasonable standard of merit or they would not have been published where they were. But all this shows, if it shows nothing else, a strong and persisting love for science, an unwearying industry in things scientific, and a reasonable amount of scientific capacity. These conclusions nobody can deny. But do men of whom these things can be said really and truly fulfil the objects for which the Royal Society was founded? Do they, or do they not promote, push forward, and extend the bounds of natural knowledge? Supposing science to be an army of conquest, are they not likely to be loyal and helpful soldiers? It is fortunate that the Royal Society has a majority of men of a different scientific calibre from the pedantic and ridiculous handful who set themselves against the election of Sir Henry Howarth last week. Perhaps one of the ridiculous may remember what Professor Huxley said of him on a well-known occasion some twelve or fourteen years ago. The young man on that occasion crossed swords with Professor Huxley and then claimed him as his friend. Mr. Huxley retorted that he did not know that he was entitled to the honour of being a friend *of* Mr. ———'s, but he had certainly tried to be a friend *to* him. Let us hope the ridiculous handful will find a person or persons in the Royal Society as competent to administer a merited rebuke as Professor Huxley.

